# Horizontally assembled green InGaN nanorod LEDs: scalable polarized surface emitting LEDs using electric-field assisted assembly

**DOI:** 10.1038/srep28312

**Published:** 2016-06-21

**Authors:** Hoo Keun Park, Seong Woong Yoon, Yun Jae Eo, Won Woo Chung, Gang Yeol Yoo, Ji Hye Oh, Keyong Nam Lee, Woong Kim, Young Rag Do

**Affiliations:** 1 Department of Chemistry, Kookmin University, Seoul 136-702, Korea; 2 Department of Materials Science and Engineering, Korea University, Seoul 136-713, Korea

## Abstract

In this study, we report the concerted fabrication process, which is easy to transform the size of active emitting area and produce polarized surface light, using the electric-field-assisted assembly for horizontally assembled many tiny nanorod LEDs between two metal electrodes. We fabricate the millions of individually separated 1D nanorod LEDs from 2D nanorod arrays using nanosphere lithography, etching and cutting process of InGaN/GaN LED structure on a flat sapphire substrate. The horizontally assembled InGaN-based nanorods LED device shows bright (~2,130 cd/m^2^) and uniform polarized (polarization ratio, ρ = ~0.61) green emissions from large area (0.7 cm × 0.6 cm) planar surface. The realization of a horizontally assembled nanorod LED device can prove the concept of an innovative idea to fabricate formable and scalable polarized surface LED lighting.

To date, many reports regarding millimeter-size LED chips have focused on improving energy efficiency levels (i.e., both internal efficiency and extraction efficiency)^1^,^2^ and reducing efficiency droop^1^,^3^ for applications to general lighting and giant outdoor displays. Inorganic InGaN LED chips are usually applied to fabricate point light sources with unpolarized emission due to the geometrical and technological limitations of millimeter- and a-few-hundred-micrometer-sized chips^4^,^5^,^6^. The inorganic InGaN LED application areas require the development of tiny manageable LEDs and a device layout that can be simply transformed into conventional and innovative LED-based devices, such as surface lighting, pixels for TV-scale indoor/mobile displays, and polarized surface light sources. Recently, advanced research on growing LED applications has found that linear polarized light sources enhance the efficiency of most present and future applications, including antiglare^7^ and anti-eyestrain lighting^8^, optical communication^9^, and LCD backlighting^10^,^11^. However, most polarized LEDs require an optical structure or an additional fabrication step, such as redesigning the epi-layer^12^,^13^, embedding periodical nanostructures^14^,^15^,^16^, or integrating polarized optical media with the LED materialsm^17^,^18^.

Recently, the development of one-dimensional (1D) GaN nanostructures has stimulated much interest because of potential improvements in luminance, internal efficiency^19^, and extraction efficiency^20^, potential mitigation of the droop problem of GaN-based LEDs^21^, and potential polarized light effects^22^ that can be utilized in various optoelectronic applications. A bottom-up approach has been demonstrated to be capable of fabricating high quality 1D GaN nanostructures by avoiding plasma damage^23^,^24^, and non-polar or semi-polar GaN nanostructures^25^,^26^. Also, in spite of the plasma damage the occurs during plasma etching, research into fabricating 1D GaN by top-down approach has been reported because of the easy to control parameters and fabrication precision for dimensions of large wafer scale^27^,^28^. Meanwhile, various assembly technologies such as layer-by-layer method^29^, transfer printing^30^,^31^, and fluidic flow-assisted^32^,^33^ and electric field-assisted techniques^34^,^35^ have been used to position 1D GaN nanowires on predefined areas or pre-patterned electrodes and to evaluate the optical, electrical, and polarized emission properties of single nanowires or vertically aligned multiple nanowires. Especially, research related to the electric field-assisted technique has been reported for 1D GaN wire transistor parts because of the need to accomplish precise positioning^34^,^36^. However, in spite of any potential precise positioning, research into 1D GaN LEDs using the electric field-assisted technique has demonstrated only the connection of single or several spot light sources, such as single 1D GaN that were fabricated using focused ion beam deposition^37^; several 1D GaN were also fabricated via dielectricphoresis^38^. In a recent trend of 1D GaN nanostructures, research into wafer-based millimeter-scale devices using 1D GaN nanostructures in optoelectronic application has demonstrated an improved engineering design. *Dai et al*. reported a multi-layer flexible LED device and a white LED device with vertical assembled m-GaN wires in polydimethlysiloxane (PDMS) at millimeter scale^39^. *Ahn et al*. demonstrated 3D heterogeneous electronics using transfer printing^40^.

In this study, we propose the concerted fabrication of individually separated GaN-based nanorod LEDs as nano-emitters and the development of a horizontally assembled nano-LED system with millions of tiny nanorods that can be evolved into planar surface lighting or direct-view displays, as well as polarized light sources. Our technology is divided into three techniques: the first is a wafer-scale technology that combines bottom-up and top-down technologies to produce individually separated nanorod LEDs with InGaN/GaN multiple quantum wells (MQW); the second is a controlled dielectrophoretic assembly technology for millions of individually separated InGaN/GaN green-emitting nanorod LEDs as interconnected arrays on pre-patterned interdigitated electrodes; the third is an advanced interconnection scheme and horizontal device layout to facilitate electrical contact and system integration. These concerted approaches allow the devices to be easily implemented in a wide range of scalable self-emissive polarized surface LED devices with large area (0.7 cm × 0.6 cm) for surface lighting or small pixel area (100 μm × 100 μm) for displays. Although this nano-LED approach cannot at this moment provide a perfect solution for the realization of highly efficient LED surface lighting and LED display using nanoscale LEDs, this concerted attempt at the creation of a concept for combining different fabrication approaches is considered to be an initial suggestion toward possible nanoscale LED devices that can be realized in the future.

## Materials and Methods

### Materials

Polystyrene (PS) nanosphere (with a diameter of 960 nm) was purchased from Interfacial Dynamics Co. and used as a mask layer for fabricating nanorod arrays. Sodium dodecyl sulfate (SDS, NaC_12_H_25_SO_4_) was obtained from Sigma-Aldrich for forming a rigid PS nanosphere monolayer. CR-7, used as the Cr etchant, was purchased from Cyantek Co. Ltd., and KOH was obtained from Daejung Chemicals & Metals Co. Ltd. for fabricating cylindrical nanorod arrays via wet-etching process of tapered nanorod arrays. HAuCl_4_ was purchased from Sigma-Aldrich for the electrochemical deposition of Au nanoparticles.

### Formation of green-emitting InGaN/GaN cylindrical nanorod arrays on sapphire substrate and individually separated nanorod LEDs

The explanations about fabrication of InGaN/GaN cylindrical nanorod array are detailed in Supplementary Information 1-1.

### Assembly and alignment of individually separated InGaN/GaN nanorod LEDs between interdigitated finger-pattern metal electrodes

The explanations about assembly and alignment of individually separated InGaN/GaN nanorod LEDs between interdigitated finger-pattern metal electrodes are detailed in Supplementary information 2-2.

### Characterization

The optical and electrical analyses about green-emitting InGaN/GaN LED are detailed in Supplementary information 3.

## Results and Discussion

**Figure 1:**
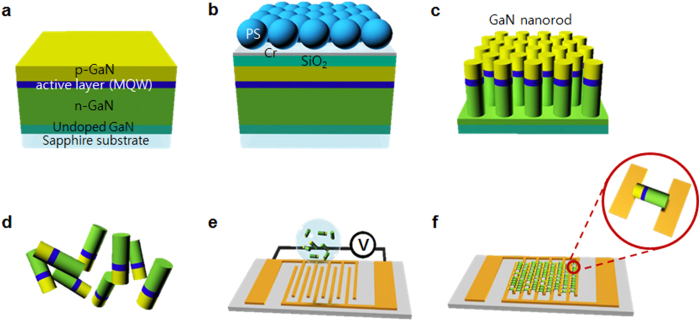
Schematic of the fabrication procedure of a triangularly patterned InGaN/GaN cylindrical nanorod LED structure, an individually separated nanorod LED, and nanorod LEDs aligned between metal electrodes. (**a**) Conventional InGaN/GaN MQW-based green LED structure on a flat sapphire substrate. (**b)** PS nanospheres in a monolayer form prepared on the Cr/SiO_2_/GaN substrate using a scooping transfer technique. (**c**) Triangularly patterned cylindrical nanorod arrays fabricated on the sapphire substrate. (**d**) Individually separated nanorod LEDs obtained by cutting the sample using a diamond knife. (**e**) Application of voltage for alignment of the individually separated nanorod LEDs between the interdigitated finger metal electrodes. (**f**) Nanorod LEDs aligned between the metal electrodes.

Successful demonstration in the present study requires a process that combines bottom-up and top-down methods for fabricating individually separated green-emitting nanorod LEDs with InGaN/GaN MQWs and an assembly method for generating a new planar green surface light with a polarization effect. Triangularly patterned two-dimensional (2D) InGaN/GaN nanorod LED structures were fabricated on a 2-inch wafer-scale GaN film (p-GaN/InGaN MQWs/n-GaN/un-doped GaN supporting film) on a sapphire substrate through a combination of self-assembled polystyrene (PS) nanosphere deposition based on the scooping transfer technique^41^,^42^,^43^,^44^ and a dry/wet double etching process^45^. The fabrication procedures of the 2D nanorod LED array structures and individually separated 1D nanorod LEDs, as well as alignment procedures of the 1D nanorod LEDs between metal electrodes, are illustrated in Fig. 1 and are described at full length in the Supporting Information 1-1, 2-1 and 2-2.

**Figure 2:**
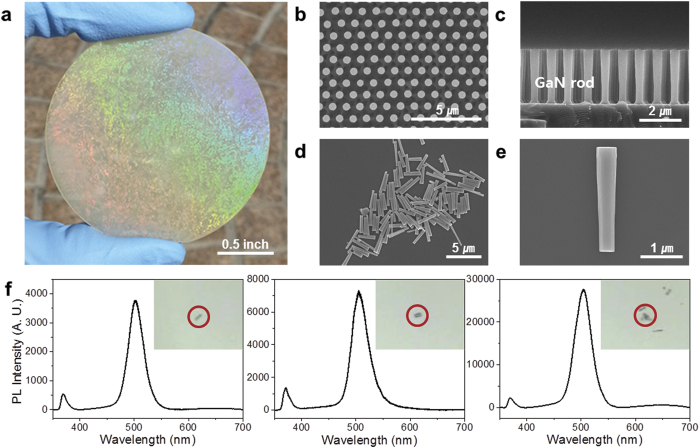
Fabrication of the individually separated InGaN/GaN cylindrical nanorod LEDs. (**a**) Triangularly patterned InGaN/GaN cylindrical nanorod array fabricated on a 2-inch wafer-scale sapphire substrate. (**b**) Top-view and (**c**) side-view SEM images of the cylindrical nanorod array with a thickness of ~2.5 μm and diameter of ~500 nm. (**d**) Top-view SEM image of the individually separated nanorod LEDs. (**e**) Magnified SEM image of a finely fabricated single nanorod LED. (**f**) PL spectra of single-, double-, and multi-nanorod LEDs at room temperature. Inset: optical images of single-, double-, and multi-nanorod LEDs.

Figure 2a provides images of the 2-inch wafer-scale GaN film with a triangularly patterned 2D InGaN/GaN cylindrical nanorod array. Figure 2b,c shows top-view and side-view scanning electron microscopy (SEM) images of the triangularly patterned 2D InGaN/GaN cylindrical nanorod array with a height of ~2.5 μm and top-side diameter of ~500 nm (aspect ratio [AR] = ~5.0) on a sapphire substrate. Individually separated nanorod LEDs were cut using a diamond knife (Fig. 2d). Figure 2e shows a finely fabricated single 1D InGaN/GaN green-emitting nanorod LED on Si substrate. These SEM results indicate that our combined fabrication process can be successfully used to uniformly fabricate millions of 1D InGaN/GaN cylindrical nanorods.

For the investigation of the optical properties of the as-grown InGaN/GaN planar sample and 2D cylindrical nanorod array sample on sapphire substrate, the photoluminescence (PL) spectra of two-typed samples were obtained at temperatures of 10 K and 300 K using a He-Cd laser (325 nm) as a excitation source (see Supplementary Information 1-2-1). Moreover, to check the blue-shift phenomenon and peak broadening for a single 1D nanorod LED itself—in respect to the PL of the 2D nanorod arrays exhibiting blue-shift and broadening—compared to that of the as-grown planar sample, the micro-photoluminescence (μ-PL) properties of individually separated 1D nanorods LEDs were observed using μ-PL measurements at room temperature. Figure 2f shows the PL spectra and optical images (inset) of individual single-, double-, and multi-nanorod LEDs dispersed on Si substrate. The PL spectra exhibited a dominant peak of similar green emission from InGaN/GaN MQW layer compared to the nanorod arrays at a temperature of 10 K (Figure S2a), and the PL intensity increased with an increasing number of 1D nanorod LEDs. The emission of the GaN layer (the bandgap and wavelength of GaN are 3.4 eV and 364 nm, respectively) exhibited a low value compared to that from the InGaN/GaN MQW layer^46^,^47^,^48^. The defect-related luminescence peak was hardly detected in the individual single-, double-, and multi-nanorod LEDs, although it was detected in the nanorod arrays at a temperature of 300 K (Figure S2b). These results confirm that individually separated 1D nanorod LEDs with InGaN/GaN MQWs are not detectably degraded by the plasma etching process and exhibit a good optical quality that leads to several types of new LED devices.

The cathodoluminescence (CL) properties of the as-grown InGaN/GaN planar sample, 2D cylindrical nanorod array sample, and individual single-nanorod sample were compared using micro-CL measurement. (See more details of CL properties in Supplementary Information 1-2-2.) The CL results of the as-grown InGaN/GaN planar sample, 2D cylindrical nanorod array sample, and individual single-nanorod sample were similar to the PL results. These results reconfirm that the single-nanorod LED was well fabricated with good optical properties using our combined process of bottom-up and top-down processes.

To investigate the structural properties of individual InGaN/GaN single-nanorod LEDs, high-resolution transmission electron microscopy (HRTEM) measurement was carried out as shown in Supplementary Information 1-2-3. The HRTEM results in Figure S5 confirm that the 1D GaN nanorod LED has a single-crystalline structure with few observable defects and is not noticeably damaged by the plasma etching process.

**Figure 3:**
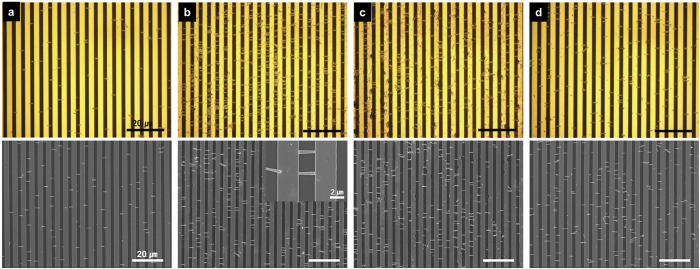
Alignment of the individually separated nanorod LEDs between the metal electrodes. Top-view optical microscope images (upper images) and SEM images (lower images) of InGaN/GaN nanorod LEDs aligned with the finger metal electrodes under various sinusoidal voltages and frequencies: (**a**) 14.0, (**b**) 17.5 and (**c**) 21.0 V_rms_ with a frequency of 950 kHz, and (**d**) 17.5 V_rms_ with a frequency of 100 kHz. The inset in (**b**) shows the magnified SEM image of a single nanorod LED.

Using two parallel metal electrodes, the dielectrophoretic assembly technology of individually separated 1D InGaN/GaN nanorod LEDs was conducted^34^,^35^. The metal electrodes (0.7 cm × 0.6 cm) consist of interdigitated finger patterns (finger width of ~3 μm and spacing of ~2.5 μm) fabricated by photolithography and Au metal lift-off process (See more details about fabrication of metal electrodes in Supplementary Information 2-1.). Figure 1e,f shows a schematic diagram of the alignment of the separated InGaN/GaN nanorod LEDs with the interdigitated finger metal electrodes under nonuniform electric fields (See more details about the dielectrophoretic assembly of individually separated 1D InGaN/GaN nanorod LEDs in Supplementary Information 2-2). Figure 3 shows top-view optical microscope images and SEM images of 1D InGaN/GaN nanorod LEDs aligned with the finger metal electrodes under various sinusoidal voltages and frequencies. Under a voltage of 14.0 V_rms_ (40 V_PP,_ peak-to-peak voltage, from −20 V to 20 V) and a frequency of 950 kHz, the nanorod LEDs were aligned between pairs of metal electrodes as shown in Fig. 3a. When the voltage increased from 14.0 V_rms_ to 17.5 V_rms_, the number density (~70.2%)—defined as the ratio of the number of aligned LEDs to the number of total LEDs (all aligned and non-aligned LEDs)—of aligned LEDs increased significantly compared with that (~16.9%) of aligned LEDs at 14.0 V_rms_ (see Fig. 3b). The presented number density values were averaged over five different locations on each sample. The metal electrodes were randomly in contact with the head (p-type GaN layer) or tail (n-type GaN layer) of the LEDs (see inset, Fig. 3b). The field emission SEM picture in the inset indicates that the nanorod LED of length ~2.5 μm and diameter ~500 nm (AR = ~5.0) successfully made insertion between or contact with the parallel metal electrodes. However, when the voltage increased to 21.0 V_rms_, the number density (~69.2%) of aligned LEDs decreased slightly and the metal electrodes were burned and destroyed by the high voltage (see Fig. 3c). Moreover, when the frequency decreased from 950 to 100 kHz at a constant voltage of 17.5 V_rms_, the number density (~20.4%) of aligned LEDs also decreased significantly (see Fig. 3d). Thus, the alignment experiments depending on the sinusoidal voltage and frequency for alignment indicate that at a voltage of 17.5 V_rms_ and a frequency of 950 kHz, the force applied to the nanorod LEDs was sufficiently strong to drive the alignment. Therefore, this dielectrophoretic assembly technology can be used to position a high density of 1D nanorod LEDs on pre-patterned electrodes; also, highly concentrated nanorod LEDs on predefined electrodes can be transformed into designed electrodes for large planar surface lighting and pixels for direct-view displays.

Another important problem with the assembled InGaN/GaN nanorod LED layout pertained to the poor interconnection between the nanorod LEDs and the interdigitated metal electrodes just after assembly. In order to realize an advanced interconnection and facilitate electrical contact between the nanorod LEDs and the metal electrodes, electrochemical deposition of Au was performed on top of the electrode and contact points between the nanorods and the electrodes. Rapid thermal annealing (RTA) was subsequently performed at 810 °C for 120 s in ambient N_2_ (See more details about electrochemical deposition and RTA processes in Supplementary Information 2–3)^49^,^50^.

**Figure 4:**
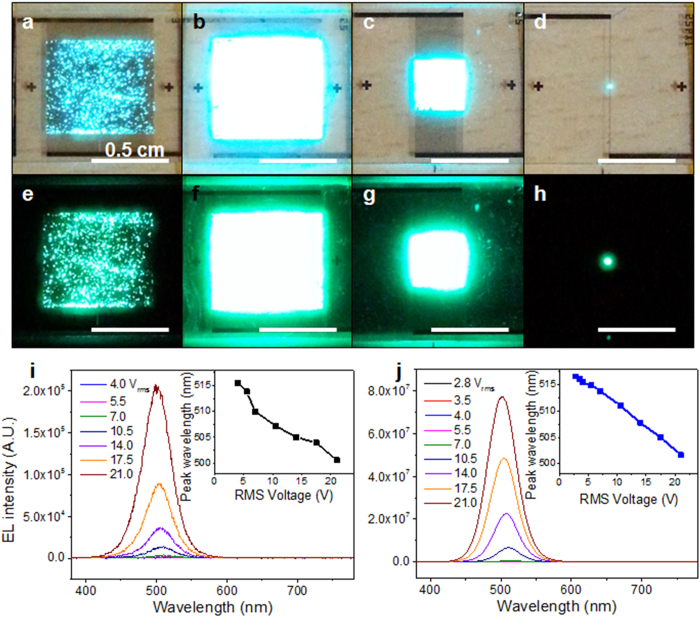
Electroluminescent properties of green-emitting InGaN/GaN nanorod LEDs aligned between the metal electrodes. Photographs of EL devices with aligned nanorod LEDs at voltages of 21.0 V_rms_ in the presence of background light (**a**) as-assembled with 0.7 cm × 0.6 cm area, (**b**) post-treated with 0.7 cm × 0.6 cm area, (**c**) post-treated with 0.3 cm × 0.3 cm area, and (**d**) post-treated with 100 *μ*m × 100 *μ*m area, and without background light (**e**) as-assembled with 0.7 cm × 0.6 cm area, (**f**) post-treated with 0.7 cm × 0.6 cm area, (**g**) post-treated with 0.3 cm × 0.3 cm area, and (**h**) post-treated with 100 *μ*m × 100 *μ*m area. EL spectra of (**i**) as-assembled EL devices under applied voltages ranging from 4.0 to 21.0 V_rms_ and (**j**) post-treated EL devices under applied voltages ranging from 2.8 to 21.0 V_rms_ (The insets in show the EL emission peak wavelength of as-assembled and post-treated EL devices as a function of injection voltage).

AC sinusoidal voltages were applied to the metal electrodes ranging from 0 to 21.0 V_rms_ at 60 Hz in order to observe the collective light emission from a large number of 1D InGaN/GaN nanorod LEDs aligned between the metal electrodes. A detectable pure green light emission of as-assembled and post-treated electroluminescence (EL) samples could be observed at different ranges. Figure 4 shows photographs of EL emissions and EL spectra of as-assembled and post-treated EL samples. Figure 4 also displays photographs of EL emissions of size-reduced EL samples (EL samples of 0.3 cm × 0.3 cm and 100 *μ*m × 100 *μ*m area) with and without background light. As the voltage of the as-assembled EL device increased, the green emission was visible to the naked eye even in the presence of background light (see Fig. 4a). Without background light, a planar surface type of bright green emission also was observed from the large area planar EL device (see Fig. 4e), although slight variations in brightness were observed by the naked eye over the entire EL device. After the Au electrodeposition and RTA processes (post-treatment), the green EL emission significantly increased compared that of the as-assembled LEDs (See Fig. 4b,f). This means that the interconnection and electrical contact were enhanced by the partial deposition and melting of the Au nanoparticles between the nanorod LEDs and the metal electrode. By reducing the contact resistance and increasing the connected sites between the nanorod LEDs and the electrodes, a detectable green light emission from the post-treated device could be observed at lower voltages (in the range of 2.8 to 3.5 V_rms_) compared to the as-assembled device (in the range of 4.0 to 5.5 V_rms_). These EL data demonstrate that the interconnection and electrical contact between the nanorod LEDs and the metal electrodes can be improved by the electrodepositing and annealing process. In addition, the EL intensity of the post-treated nanorod LED devices, measured according to the increase of voltage, was much higher than that of the as-assembled device (see Fig. 4i,j). Under a voltage of 21.0 V_rms_, the EL intensity of the post-treated sample increased by a factor of approximately 371. The insets in Fig. 4i,j show the corresponding EL emission peak wavelength as a function of injection voltage. The emission peak wavelengths of the as-assembled and post-treated EL samples exhibited blue-shifts of approximately 14.9 and 13.8 nm, respectively, with an increase of voltage from 4.0 to 21.0 V_rms_. These blue-shifts of EL emission are usually observed in conventional InGaN-based planar LEDs because of the screening effect of the strain-induced quantum-confined Stark effect (QCSE)^51^. The pure green emission of the collective EL from the many InGaN/GaN nanorod LEDs, without any defect-related emissions, confirms that the optical quality levels are not detectably damaged by any of the combined fabrication and assembly processes, including the plasma etching process and AC assembly process. Moreover, the EL emission figures of the size-reduced EL samples shown in Fig. 4c,d,g,h demonstrate that highly concentrated nanorod LEDs can be used as a nano-emitter source from a large area of surface lighting to a pixel array of direct-view displays.

**Figure 5:**
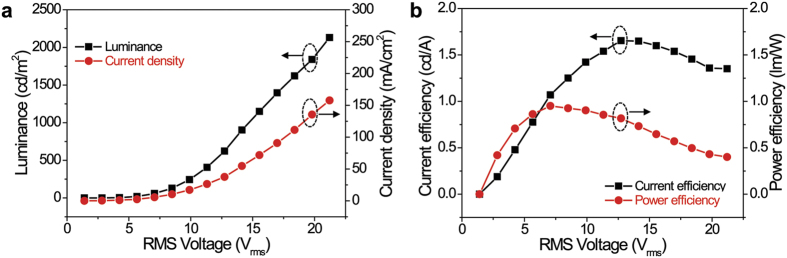
AC voltage-dependent variations of electroluminescent properties for post-treated green-emitting InGaN/GaN nanorod LEDs aligned between the metal electrodes with 0.7 cm × 0.6 cm area. (**a**) I-V and V-L and (**b**) voltage-EL luminous efficacy curve of EL emissions for post-treated EL sample.

Figure 5 presents AC voltage-dependent variations of luminance, current density, current efficiency (CE), and power efficiency (PE) of the post-treated nanorod EL device. The post-treated nanorod EL device exhibited a maximum luminance of ~2,130 cd/m^2^, current efficiency of ~1.65 cd/A, and power efficiency of ~0.95 lm/W. The current efficiency and power efficiency of the post-treated nanorod EL device was significantly low compared to the luminance of the commercial device. This is because the post-treated nanorod EL device still had high current leakage even after our interconnection processes. This high current leakage is considered to be caused by several reasons, such as incomplete purification of 1D nanorods (size fluctuations, incomplete nanorods, etc.), various defects on the electrodes (short-electrode, dust, photoresist debris, etc.), misaligned nanorods on electrodes, and still-unconnected nanorods with electrodes. However, a planar surface green EL emission from a large number of individually separated luminous InGaN/GaN nanorod LEDs has not been reported in any previously published work. To the best of our knowledge, this is the first demonstration of such collective surface green EL emission from many InGaN/GaN nanorod LEDs aligned between metal electrodes with a large planar area. Our approach indicates that spatially uniform mixing of tiny lights emitted from nanorod LEDs can be realized as an efficient planar surface source if most of the assembled nanorod LEDs are interconnected with electrodes and turned on at a low applied voltage. Therefore, in the near future, more elaborate and controlled assembled experiments under a better clean room environment are required to fabricate bright, efficient, uniform, and stable nanorod LEDs for application to various direct-emissive LED devices, highly efficient planar surface lighting, or direct-view indoor displays.

**Figure 6:**
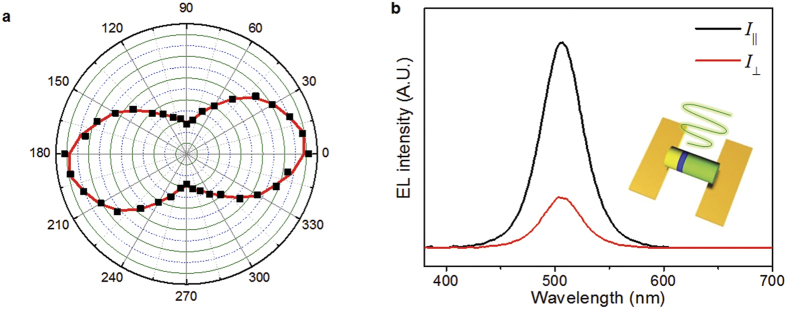
(**a**) Polar plots of the EL intensity as a function of polarizer angle for the post-treated nanorod EL device. (**b**) EL spectra of polarized emissions from the post-treated nanorod EL device.

The polarization of the post-treated nanorod EL device was also investigated by placing a linear polarizer in front of the spectrophotometer (or EL device). Figure 6a shows the polar plots of the EL intensity as a function of polarizer angle for the post-treated nanorod EL device. The EL emission is linearly polarized with the polarization ratio—defined as ρ = (*I*_∥_ − *I*_⊥_)/(*I*_∥_ + *I*_⊥_)—of ~0.61 along the *c* axis of the nanorods within a horizontally assembled nanorod EL device, where *I*_∥_ and *I*_⊥_ are the emission intensities parallel and perpendicular to the *c* axis of the nanorods, respectively. Linear polarized light emission is generated from the nanorod LEDs due to the rod shaped LEDs with 5:1 aspect ratio and highly ordered, horizontally aligned nanorod LEDs. This result is further verified by the EL spectra, as shown in Fig. 6b. The EL spectra clearly shows that the EL emission from the post-treated nanorod EL device is polarized to a considerable degree. This indicates that the observed EL anisotropy results from the oriented nanorod LEDs parallel to the nanorod axis, which is favorable for future applications requiring polarized surface light emission from horizontally assembled nanorod LEDs, leading to antiglare surface lighting, optical communication of polarized light, and photo-selective phototherapy planar patch as well as backlighting applications in liquid crystal displays. To further improve the polarization ratio, it is necessary to study the structural effect (aspect ratio of nanorods and density of aligned nanorods, for example) and any other variables on the degree of polarized light emitted from the nanorod LED-based surface lighting.

## Conclusion

In this paper, we present and demonstrate a facile and concerted method for fabricating a nano-LED device system with many horizontally assembled InGaN/GaN nanorods that can be evolved into designed layouts for planar surface lighting or pixels for direct-view indoor displays as well as polarized surface light sources. Triangularly patterned 2D InGaN/GaN nanorod arrays and millions of individually separated 1D nanorod LEDs were fabricated through a combined approach of self-assembled PS nanosphere deposition, dry/wet double etching, and direct-cutting processes. Using dielectrophoretic assembly technology, we developed a horizontally assembled nanorod-based EL device with collective planar green EL emission from many InGaN/GaN nanorod LEDs aligned between interdigitated metal electrodes with a large area (0.7 cm × 0.6 cm planar area) for surface lighting and a small pixel area (100 μm × 100 μm) for TV-scale direct-view displays. This nanorod EL device exhibits maximum luminance of ~2,130 cd/m^2^ and current efficiency of ~1.65 cd/A, and power efficiency of ~0.95 lm/W after post-treatment processes. This low electrical performance could be improved by more elaborate and controlled experiments under a better cleanroom environment that fabricate millions of tiny nanorods with high purity, assemble the nanorods between electrodes with improved density, and interconnect nanorods and electrodes with enhanced connectivity. In addition, a meaningful polarization ratio of ~0.61 is observed from the nanorod-based surface EL device. Although more studies will be needed to enhance the optical and electrical properties of the nanorod LEDs by improvement of the contact properties between the nanorod LEDs and the finger metal electrodes, the horizontally assembled nanorod LEDs and their planar shaped and polarized surface emission, as demonstrated in this work, can potentially be used in a wide range of self-emissive surface LED applications, such as scalable and formable surface lighting, pixel arrays for TV-scale indoor emissive displays, polarized surface lighting, and other innovative applications.

## Additional Information

**How to cite this article**: Park, H. K. *et al*. Horizontally assembled green InGaN nanorod LEDs: scalable polarized surface emitting LEDs using electric-field assisted assembly. *Sci. Rep.* **6**, 28312; doi: 10.1038/srep28312 (2016).

## Supplementary Material

Supplementary Information
