# Supplementary material for: Horizontally assembled green InGaN nanorod LEDs: scalable polarized surface emitting LEDs using electric-field assisted assembly
Source: Sci Rep. 2016 Jun 21;6:28312. doi: 10.1038/srep28312 (PMC4915009; doi:10.1038/srep28312)
Supplement: Supplementary Information [file srep28312-s1.pdf]

Supplementary Information (SI)

Horizontally assembled green InGaN nanorod LEDs: scalable polarized surface emitting LEDs using electric-field assisted assembly

Hoo Keun Park[a], Seong Woong Yoon[a], Yun Jae Eo[a], Won Woo Chung[a], Gang Yeol Yoo[b], Ji Hye Oh[a], Keyong Nam Lee[a], Woong Kim[b], and Young Rag Do[a,*]

[a]Department of Chemistry, Kookmin University, Seoul 136-702, Korea
[b]Department of Materials Science and Engineering, Korea University, Seoul 136-713, Korea

*To whom correspondence should be addressed. E-mail: yrdo@kookmin.ac.kr

Supplementary Information 1

SI 1. Fabrication and characterization of triangularly patterned green-emitting InGaN/GaN cylindrical nanorod arrays on sapphire substrates and individually separated nanorod LEDs

SI 1-1. Fabrication of triangularly patterned green-emitting InGaN/GaN cylindrical nanorod arrays and individually separated nanorod LEDs

Figure S1 presents the schematic and side-view scanning electron microscopy (SEM) images of the fabrication procedures of the triangularly patterned InGaN/GaN cylindrical nanorod arrays on flat sapphire substrates. Conventional InGaN/GaN MQW-based green LED structures on a flat sapphire substrate were used to prepare the cylindrical nanorod arrays. From the bottom to the top, the LED structure consisted of a 3.0 μm-thick unintentionally doped GaN layer grown on a flat sapphire substrate, a 4 μm-thick n-type GaN layer (n-GaN), a region with five periods of InGaN/GaN MQWs, and a 0.2 μm-thick p-type GaN layer (p-GaN) (**Figure S1a**). A Cr layer (~150 nm thick) and a SiO_2 layer (~1.7 μm thick) were deposited on the InGaN/GaN film-coated sapphire substrate via thermal evaporation and plasma-enhanced chemical vapor deposition (PECVD), respectively (**Figure S1b**). A monolayer of triangularly patterned PS nanospheres (diameter: 960 nm) was prepared in a water solution and coated on the Cr/SiO_2/GaN substrate using a scooping transfer technique (F**igure S1c**). The PS nanospheres were thinned using an ashing process using O_2 plasma. Next, a Cl_2-based reactive ion etching (RIE) process was performed in order to transfer the pattern to the underlying Cr layer (**Figure S1d**). The Cr nanodot array patterns appeared in the Cr layer after the PS nanospheres were removed via sonication in chloroform (**Figure S1e**). Then, the nanodot mask was transferred to the SiO_2 layer via a CF_4-based RIE on the SiO_2/GaN substrate. The residual Cr nanodot mask was removed using CR-7SK (Cr etchant). Subsequently, triangularly patterned SiO_2 nanorod arrays were formed on top of the InGaN/GaN substrate (**Figure S1f**). With the prepared SiO_2 nanorod arrays as the etching mask, triangularly patterned InGaN/GaN tapered nanorod arrays with a thickness of ~2.5 μm and diameter of ~600 nm were fabricated on the sapphire substrate through the ICP etching process using a Cl_2/BCl_3/N_2 gas mixture (**Figure S1g**). In general, a GaN nanorod array fabricated using a top-down method exhibits surface defects on the nanorod sidewalls due to the high-energy ion bombardment during the plasma etching process[S1-S3]. In order to mitigate this problem

and avoid performance degradation of the etched GaN nanorod LEDs, a KOH-based wet etching process (1 mol/L) was performed for 5 min[S3, S4]. After the KOH-based wet etching process, InGaN/GaN cylindrical nanorod arrays with a thickness of ~2.5 μm and a diameter of ~500 nm were fabricated. The remainder of the SiO_2 nanorod arrays were removed through dipping the sample into a buffered oxide etch (BOE) solution. Finally, triangularly patterned InGaN/GaN cylindrical nanorod arrays were obtained on the sapphire substrate (**Figure S1h**). Millions of individually separated InGaN/GaN nanorod LEDs were obtained through cutting the nanorod arrays in acetone using a diamond knife.

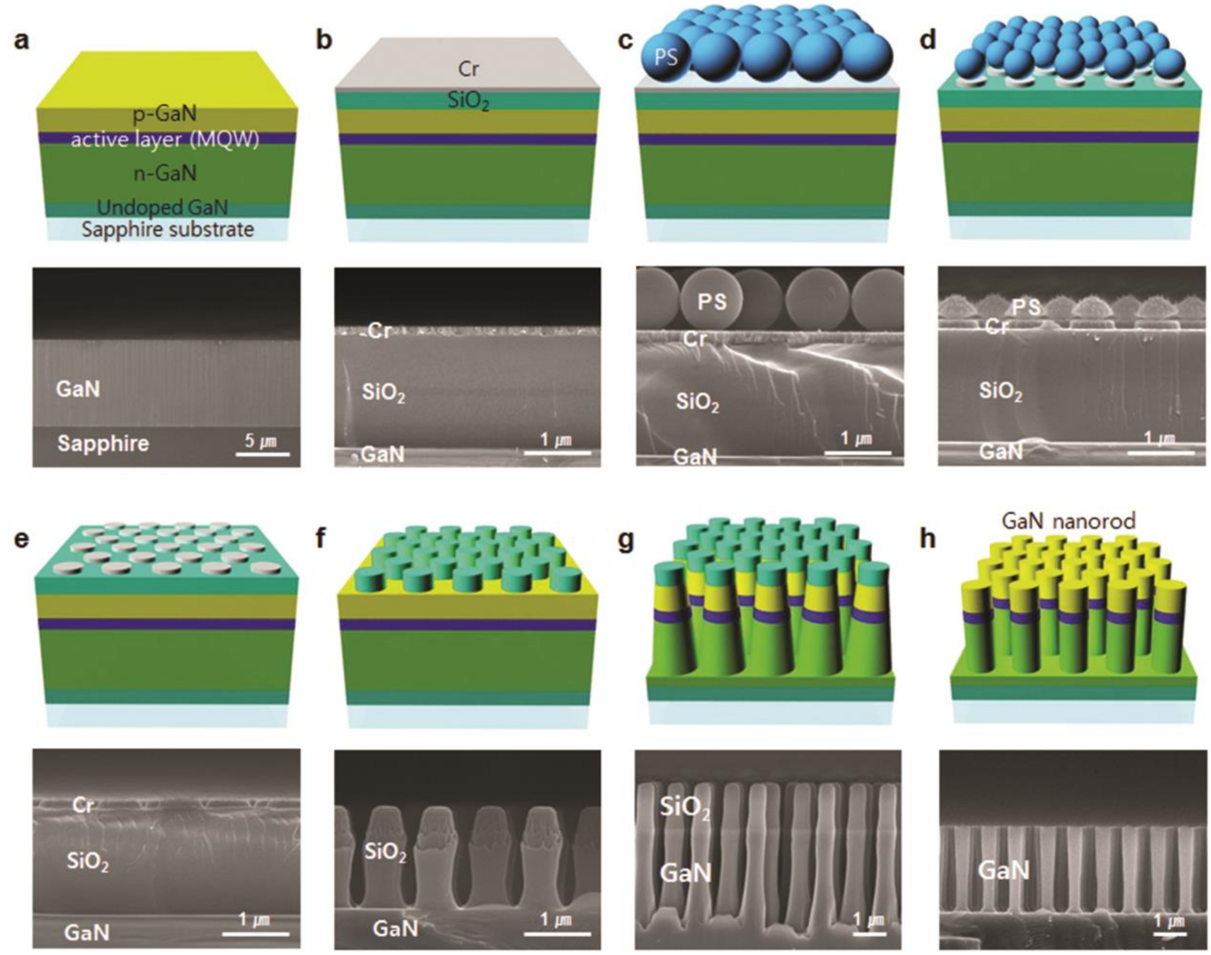

Figure S1. Schematic and side-view SEM images of the fabrication procedures of triangularly patterned InGaN/GaN cylindrical nanorod arrays on a flat sapphire substrate. **a**, Conventional InGaN/GaN MQW-based green LED structure on the flat sapphire substrate. **b**, Cr and SiO_2 layers deposited on the GaN film-coated sapphire substrate. **c**, Triangularly patterned PS nanospheres in monolayer form prepared on the Cr/SiO_2/GaN substrate. **d**, PS nanosphere monolayer thinned via an ashing process using O_2 plasma and a Cr nanodot layer prepared using a Cl_2-based RIE process. **e**, Cr nanodot layer formed after the removal of the thinned PS monolayer. **f**, Triangularly patterned SiO_2 nanorod layer fabricated using a CF_4-based RIE process, following which the Cr nanodot layer was removed. **g**, Triangularly patterned SiO_2/GaN tapered nanorod arrays fabricated on the sapphire substrate through the ICP etching process. **h**, InGaN/GaN cylindrical nanorod arrays fabricated on the sapphire substrate after the KOH wet etching process and removal of the SiO_2 nanorod layer.

SI 1-2. Characterization of triangularly patterned green-emitting InGaN/GaN cylindrical nanorod arrays and individually separated nanorod LEDs

SI 1-2-1. Photoluminescence (PL) of InGaN/GaN cylindrical nanorod arrays

Figure S2 presents the photoluminescence (PL) spectra of the as-grown InGaN/GaN planar sample and cylindrical nanorod array sample on a sapphire substrate at temperatures of 10 K and 300 K using a He-Cd laser (325 nm). The PL emission peaks of the planar and nanorod samples were 537.5 and 512.7 nm at a temperature of 10 K, and 532.6 and 516.6 nm at a temperature of 300 K, respectively. The blue-shift of the emission peaks from the nanorod sample compared with the planar sample could results from the partial reduction of the piezoelectric field due to the strain release in the nanorod. It is well known that, for InGaN/GaN MQW structures grown on the c-axis, a strong strain-induced piezoelectric polarization field exists inside the structure, which causes the band bending and red shift of the spectrum, known as the quantum-confined Stark effect (QCSE)[S5-S7]. In order to reduce the QCSE, the residual strain in the epitaxial layer that results from the fabrication in the nanorod structure must be decreased, which generates a blue-shift in the emission peak[S6-S10]. In addition, the PL emission peak of the nanorod sample at a temperature of 300 K was broadened compared with the nanorod sample at a temperature of 10 K, due to the existence of defect-related luminescence from the nanorod substrate due to the plasma etching process[S3]. The PL intensities of the nanorod sample were enhanced by factors of approximately 6.0 and 5.2 compared with the planar sample at temperatures of 10 K and 300 K, respectively. The enhanced PL intensity can be attributed to an increase in the internal quantum efficiency (IQE) that results from the partial elimination of the QCSE and to a reduction in the total internal reflection (TIR) due to the fabrication into the nanorod structure, which provides a larger surface area and increases the light extraction efficiency[S8, S10-S13].

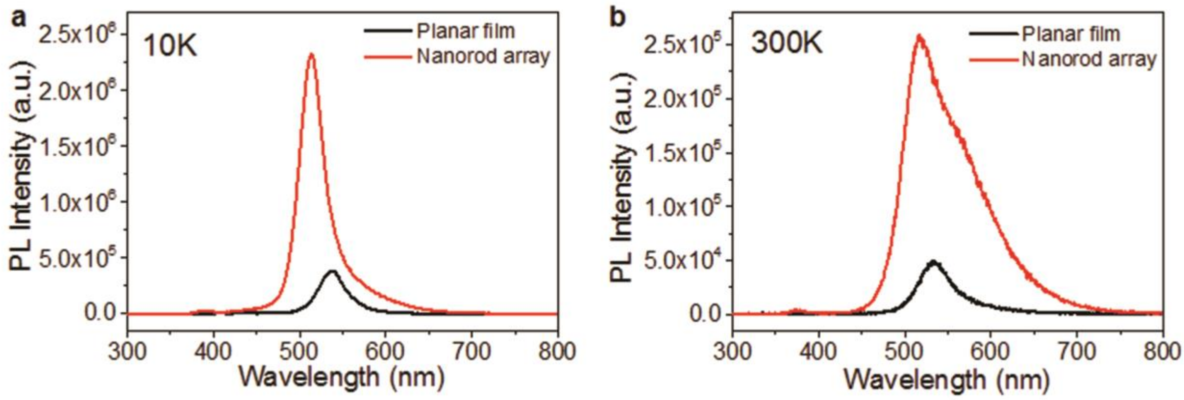

Figure S2. Photoluminescence (PL) spectra of the as-grown InGaN/GaN planar sample and cylindrical nanorod array sample on a sapphire substrate at temperatures of **a**, 10 K and **b**, 300 K using a He-Cd laser (325 nm).

SI 1-2-2. Cathodoluminescence (CL) of InGaN/GaN cylindrical nanorod arrays and individually separated single-nanorod LEDs

Figure S3 presents SEM images of the as-grown InGaN/GaN planar sample and cylindrical nanorod array sample on the sapphire substrate, their room temperature panchromatic CL emission, and their CL spectrum. From the SEM and CL images, it can be seen that the InGaN/GaN MQW region in the nanorod array sample is brighter compared with that in the planar sample due to the nanofabrication process. This result is consistent with the CL spectra in **Figures S3c** and **S3f**. The CL spectra indicate that the CL intensity from the InGaN/GaN MQW in the nanorod array was enhanced compared with the planar sample, although the CL peak from the InGaN/GaN MQW, in common with the PL peak at a temperature of 300 K, broadened due to the existence of defect-related luminescence from the nanorod substrate in the plasma etching process.

Figure S4 presents a SEM image of an individual single-nanorod LED, its room temperature panchromatic CL emission, and its CL spectrum. The diameter of the nanorod LED including the p-GaN top, InGaN/GaN MQWs, and n-GaN bottom were uniform (**Figure S4a**). From the CL mapping image in **Figure S4b**, it can be observed that the InGaN/GaN MQW region was significantly brighter when compared with the n-GaN region. In addition, the CL spectrum of the InGaN/GaN MQW region exhibited a stronger emission than the p-GaN and n-GaN regions as depicted in **Figure S4c**. The strong band edge emission peaks of InGaN/GaN and GaN were observed at 498 and 366 nm, respectively. Other additional peaks were not detected and the defect-related luminescence peak was also seldom detected. These results demonstrate that the single-nanorod was fabricated with good optical properties using the proposed process.

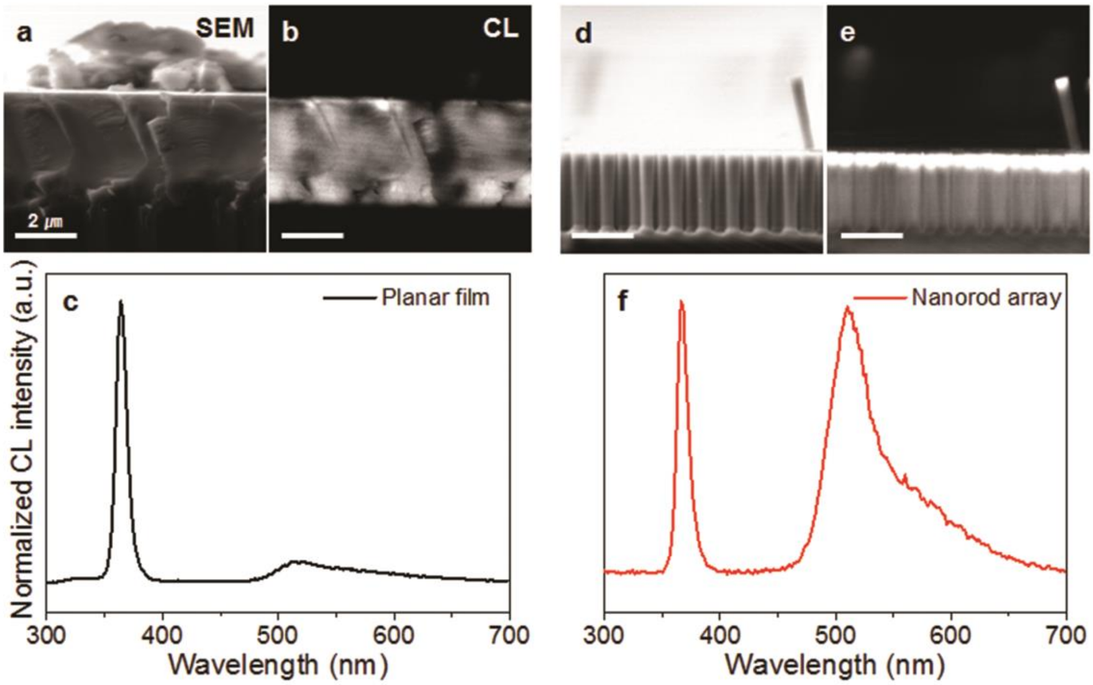

Figure S3. (**a** and **d**) SEM image, (**b** and **e**) room temperature panchromatic CL emission, and (**c** and **f**) CL spectrum of the as-grown InGaN/GaN planar sample and cylindrical nanorod array sample on a sapphire substrate.

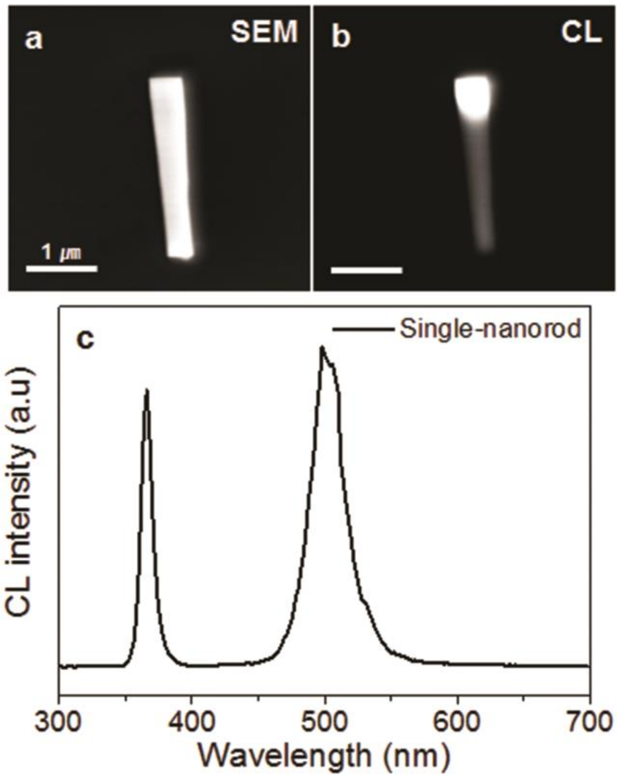

Figure S4. **a**, SEM image of an individual single-nanorod LED, **b**, its room temperature panchromatic CL emission, and **c**, its CL spectrum.

SI 1-2-3. Transmission electron microscopy (TEM) of individually separated single-nanorod LED

Figure S5 presents the low-resolution and high-resolution TEM images, and their corresponding selected-area electron diffraction (SAED) patterns, of the individually separated single-nanorod LED. As seen in **Figures S5a** and **S5b**, significant crystal defects such as dislocations were not found in the MQW or GaN regions, even though the nanorod LED was fabricated using a top-down method. From the high-resolution TEM image and the corresponding selected-area electron diffraction (SAED) pattern of the nanorod LED in **Figures S5c** and **S5d**, the lattice spacing between the adjacent planes, which corresponds to the d-spacing of the GaN [0001] planes, was determined to be approximately 0.52 nm. In addition, this demonstrates that the [0001] direction was a common growth direction for the planar GaN layer with InGaN/GaN MQWs. These results confirm that the GaN nanorod LED has a single-crystalline structure with few observable defects and is not noticeably damaged within the HR-TEM analysis range.

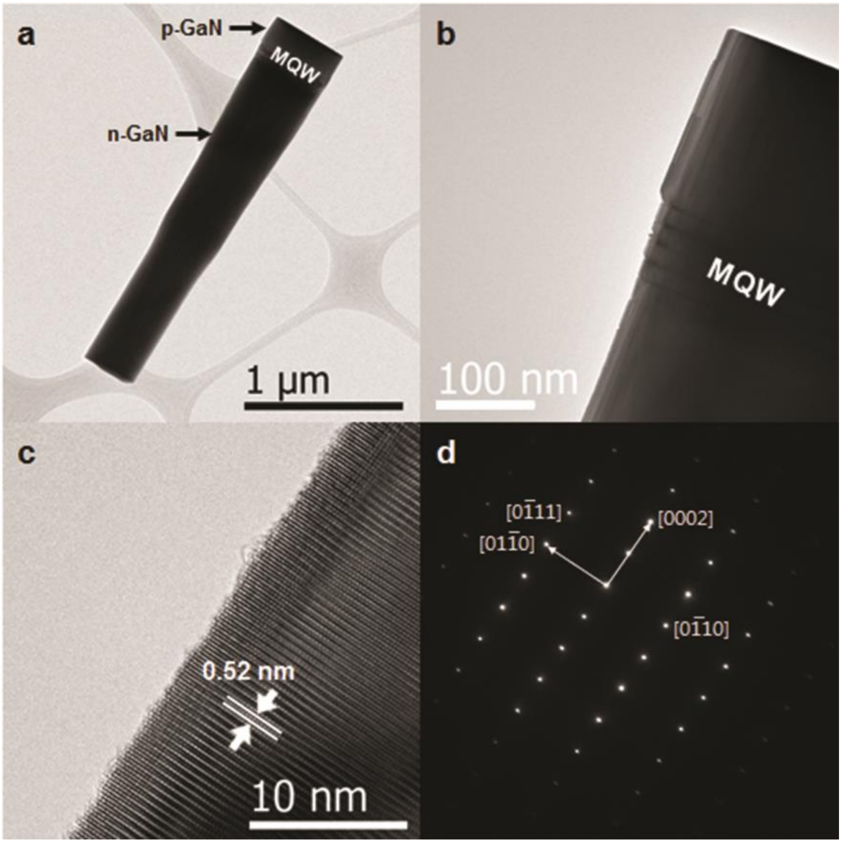

Figure S5. **a**, Low-resolution TEM image, (**b** and **c**) high-resolution TEM images, and **d**, corresponding selected-area electron diffraction (SAED) pattern of the individually separated single-nanorod LED.

Supplementary Information 2

SI 2. Assembly, alignment, and characterization of individually separated InGaN/GaN nanorod LEDs between interdigitated finger-pattern metal electrodes

SI 2-1. Fabrication of interdigitated finger-pattern metal electrodes

Figure S6 presents the schematics of the fabrication of the inter-digitated finger-pattern metal electrodes. The bare glass substrate was ultrasonically cleaned in acetone, ethanol, and de-ionized water for 10 min in each solution and then dried with N_2 gas (**Figure S6a**). The negative PR (N-PR) was coated on the bare glass substrate via spin coating (500 rpm for 5 sec and 3500 rpm for 35 sec) (**Figure S6b**). Using the photolithography process through an interdigitated Cr mask, the inter-digitated finger PR-pattern was fabricated on the glass substrate (**Figures S6c-S6e**). Then, the Ti/Au metal film was deposited on the interdigitated finger PR-patterned glass substrate via E-beam evaporation (**Figure S6f**). Finally, after the metal lift-off via sonication in acetone, the interdigitated finger-pattern metal electrodes were obtained on the glass substrate (**Figures S6g and S6h**).

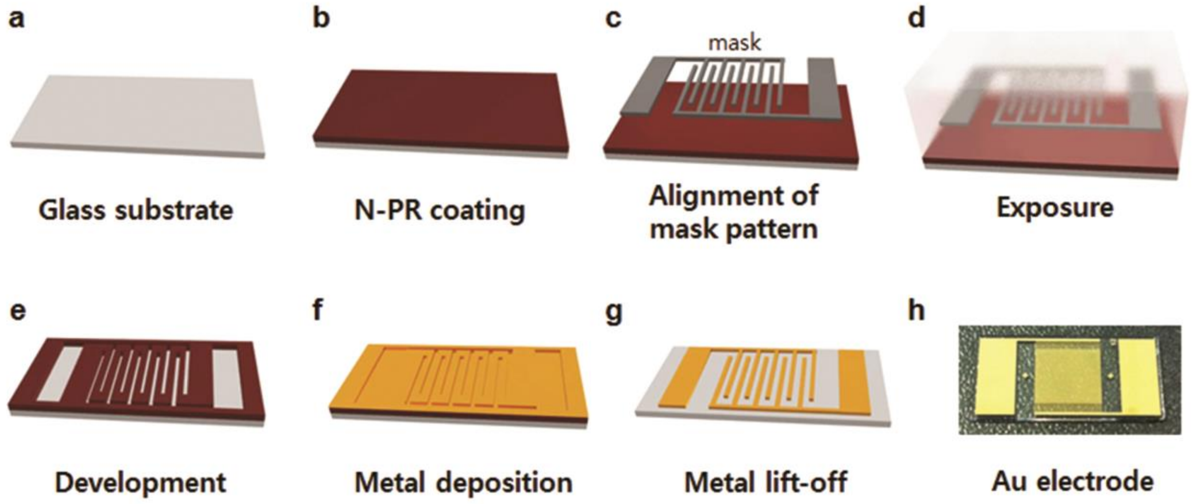

Figure S6. Schematics of the fabrication of the inter-digitated finger-pattern metal electrodes. **a**, Bare glass substrate. **b**, Negative PR (N-PR) coated on the glass substrate via spin coating. **c**, Alignment step of inter-digitated finger-patterned mask and PR-coated glass substrate. **d**, Exposure step using UV light. **e**, Development step for removal of the unexposed N-PR on the glass substrate. **f**, Deposition of the metal film (Ti/Au) on the interdigitated finger PR-patterned glass substrate via E-beam evaporation. **g**, Interdigitated finger-pattern metal electrodes fabricated on the glass substrate after the metal lift-off step. **h**, Image of the interdigitated finger-pattern Au metal electrodes.

SI 2-2. Assembly and alignment of individually separated InGaN/GaN nanorod LEDs between interdigitated finger-pattern metal electrodes

The individually separated green-emitting InGaN/GaN nanorod LEDs were suspended in acetone, which served as the transport medium during the process. Because acetone has high dielectric constant and electrical conductivity, nanorod LEDs can be effectively aligned. Also, because it has low boiling point and viscosity, acetone exhibits no coffee ring effect. A few droplets of colloidal suspension of the nanorod LEDs were dispersed onto the metal electrodes using a micropipette. An electric field was applied with sinusoidal voltages ranging from 14.0 V_{rms} to 21.0 V_{rms} and frequencies ranging from 100 kHz to 950 kHz. After the suspension was completely dried, the electric field was stopped, leaving the nanorod LEDs assembled and aligned between the metal electrodes.

SI 2-3. Realization of advanced interconnection and improvement of electrical contact between horizontally assembled InGaN/GaN nanorod LEDs and metal electrodes

Figure S7 presents the schematic of the electrochemical deposition of Au and the RTA processes for improving the interconnection, as well as the SEM images. The SEM images in **Figures S7b and S7c** clearly indicate that the Au nanoparticles were well coated on the Ti/Au electrodes and partially filled the contact points between the nanorod and electrode. **Figure S8** presents the current density characteristics observed from the green EL devices before and after the electrodepositing and annealing process, under AC sinusoidal voltage conditions (from 1.4 to 21.0 V_{rms}). This result exhibits improvements in the electrical contact behavior due to the electrodepositing and annealing, thus indicating that the post interconnecting process reduced the current leakage of the metal contact to the nanorod LEDs.

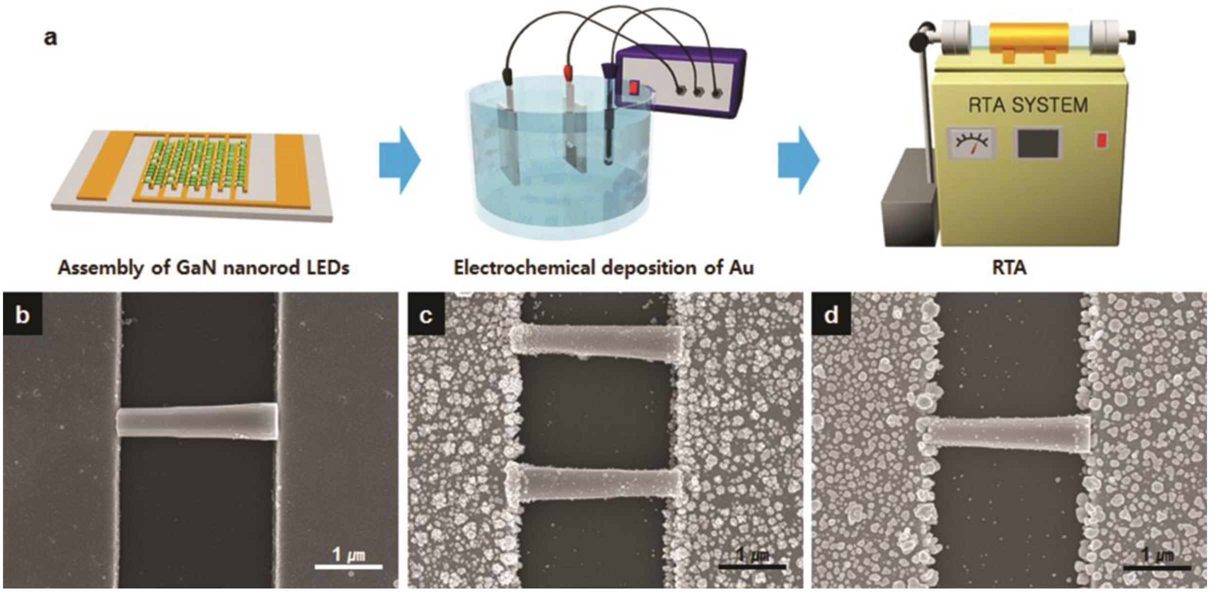

Figure S7. **a**, Schematic of the electrochemical deposition of Au and RTA processes for improving the interconnection between the nanorod LEDs and interdigitated metal electrodes after assembling; SEM images of **b**, the nanorod LED aligned between the metal electrodes, **c**, the nanorod LEDs with Au nanoparticles that were coated on the Ti/Au electrodes and partially filled into contact points between the nanorod and electrode, and **d**, the nanorod LED after the RTA annealing process.

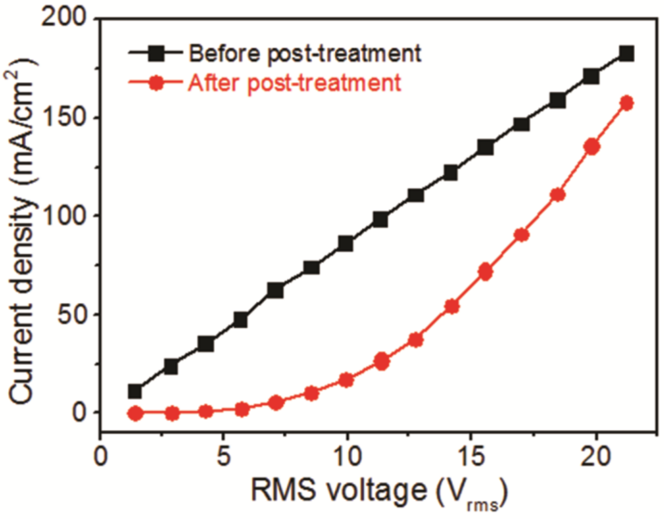

Figure S8. Current density characteristics observed from the green EL devices before and after the electrodepositing and annealing process under AC sinusoidal voltage conditions (from 1.4 to 21.0 V_{rms}).

Supplementary Information 3

SI 3. Experimental Section

SI 3-1. SEM and TEM analysis

The SEM images of the InGaN/GaN tapered nanorod arrays and cylindrical nanorod arrays on the sapphire substrates, the individually separated nanorod LEDs, and the nanorod LEDs aligned between the metal electrodes were obtained using a field emission-scanning electron microscope (FE-SEM, JSM 7401F).

The TEM specimen of the InGaN/GaN cylindrical nanorod LEDs was prepared through dispersing a colloidal suspension of the sample in acetone on a copper grid. A 200 kV field emission transmission electron microscope (FETEM, JEM-2100F) was used for the electron diffraction and for the low- and high-resolution imaging.

SI 3-2. Optical and electrical measurements

The μ-PL measurements were performed for single-, double-, and multi-nanorod LEDs at room temperature using a He-Cd laser with a wavelength of 325 nm and a CCD with a grating of 1800 gr/mm. The temperature-dependent PL peaks for the as-grown InGaN/GaN planar sample and the cylindrical nanorod array sample on sapphire substrates were measured at temperatures of 10 K and 300 K using a 325 nm He-Cd laser with an excitation power of 18 mW. The SEM-CL images and spectra for the as-grown InGaN/GaN planar sample, the cylindrical nanorod array sample, and the individual single-nanorod sample were obtained via SEM-CL measurement (Hitachi S-4300 with Gatan CL monochromator) with an electron beam of 15 kV acceleration voltage at room temperature. The optical image of the nanorod LEDs aligned between the finger-patterned metal electrodes was measured using an optical microscopy (PSI 4RT, PSI Co., Ltd., Korea).

The EL images and videos of the as-assembled and post-treated EL devices were obtained using a Sony camera (NEX-5, Sony, Japan); the EL spectra and voltage-dependent variations of the luminance, current density, current efficiency, and power efficiency for the nanorod EL device were recorded using a spectrophotometer (Model Darsa II, PSI Co., Ltd., Korea), an AC power supply (Handyscope HS5, Tie Pie Engineering, The Netherlands), a luminance colorimeter (BM7, Topcon Technohouse Co., Japan), and a digital multimeter (Model 2001, Keithley Instruments Inc., USA).

References

S1. Choi, H. W., Chua, S. J., Raman, A., Pan, J. S. & Wee A. T. S. Plasma-induced damage to *n*-type GaN. *Appl. Phys. Lett.* **77**, 1795 (2000).

S2. Oder, T. N., Kim, K. H., Lin, J. Y. & Jiang, H. X. III-nitride blue and ultraviolet photonic crystal light emitting diodes. *Appl. Phys. Lett.* **84**, 466 (2004).

S3. Li, Q. *et al.* Optical performance of top-down fabricated InGaN/GaN nanorod light emitting diode arrays. *Opt. Express* **19**, 25528-25534 (2011).

S4. Park, H., Baik, K. H., Kim, J., Ren, F. & Pearton, S. J. A facile method for highly uniform GaN-based nanorod light-emitting diodes with InGaN/GaN multi-quantum-wells. *Opt. Express* **21**, 12908-12913 (2013).

S5. Chen, H.-S. *et al.* Strain relaxation and quantum confinement in InGaN/GaN nanoposts. *Nanotechnology* **17**, 1454-1458 (2006).

S6. Wu, Y.-R., Chiu, C., Chang, C.-Y., Yu, P. & Kuo, H.-C. Size-dependent strain relaxation and optical characteristics of InGaN/GaN nanorod LEDs. *IEEE J. Sel. Top. Quantum Electron.* **15**, 1226-1233 (2009).

S7. Bai, J., Wang, Q. & Wang, T. Greatly enhanced performance of InGaN/GaN nanorod light emitting diodes. *Phys. Status Solidi A* **209**, 477-480 (2012).

S8. Chiu, C. H. *et al.* Fabrication of InGaN/GaN nanorod light-emitting diodes with self-assembled Ni metal islands. *Nanotechnology* **18**, 445201 (2007).

S9. Wang, Q., Bai, J., Gong, Y. P. & Wang, T. Influence of strain relaxation on the optical properties of InGaN/GaN multiple quantum well nanorods. *J. Phys. D: Appl. Phys.* **44**, 395102 (2011).

S10. Bai, J., Wang, Q. & Wang, T. Characterization of InGaN-based nanorod light emitting diodes with different indium compositions. *J. Appl. Phys.* **111**, 113103 (2012).

S11. Kim, H.-M. *et al.* High-brightness light emitting diodes using dislocation-free indium gallium nitride/gallium nitride multiquantum-well nanorod arrays. *Nano Lett.* **4**, 1059-1062 (2004)

S12. Huang, H. W. *et al.* Fabrication and photoluminescence of InGaN-based nanorods fabricated by plasma etching with nanoscale nickel metal islands. *J. Vac. Sci. Technol. B* **24**, 1909 (2006).

S13. Kim, S. H. *et al.* An improvement of light extraction efficiency for GaN-based light emitting diodes by selective etched nanorods in periodic microholes. *Opt. Express* **21**, 7125-7130 (2013).
